# Patterns of home- and community‐based services in older adults with dementia: an analysis of the long‐term care system in Taiwan

**DOI:** 10.1186/s12877-021-02231-9

**Published:** 2021-05-01

**Authors:** Wen-Fu Wang, Yung-Yu Su, Kai-Ming Jhang, Chun-Min Chen

**Affiliations:** 1 Department of Neurology, Changhua Christian Hospital, Changhua, Taiwan; 2 Department of Holistic Wellness, Ming Dao University, Changhua, Taiwan; 3 Department of Long Term Care, National Quemoy University, Kinmen, Taiwan; 4 Big Data Center, Changhua Christian Hospital, No. 135, Nanhsiao Street, 500-06 Changhua City, Changhua County Taiwan

**Keywords:** Dementia, Long‐term care, HCBS, Utilisation, Disability

## Abstract

**Background:**

Understanding the specific characteristics of the patients with dementia is essential in developing services required to meet their needs. The purpose of this study was to investigate the patterns of use of home- and community-based services (HCBS) by patients with dementia and the factors influencing the utilisation of these services.

**Methods:**

We analysed a dataset of patients with dementia receiving long-term care at a medical centre. All participating patients were required to complete a structured interview form every 6 months to assess their need for service utilisation. Between 2015 and 2018, a total of 822 patients fulfilled the criteria for dementia, and 737 people had assessment records, of whom 244 had used social services. Robust Poisson regression analyses were performed to estimate HCBS usage and the factors influencing service utilisation.

**Results:**

The overall service utilisation rate was 33 %. Most patients used only one service, and assistive devices were used as the main service. Regarding the factors influencing service use, dementia concomitant with dependency increased the use of HCBS. These results suggest that patients with mild dependency might prefer to use community support services, whereas those with moderate to severe dependency being more likely to utilise in-home care services.

**Conclusions:**

This study provides empirical evidence regarding the use of long-term care resources by patients with dementia in the community. Providing customised HCBS, rather than a non-specialised service assumed to be suitable for every patient, is essential for ensuring good patient care. In addition, attention needs to be paid to patients with care needs who do not use HCBS.

## Background

Population ageing is one of the major issues affecting all developed countries including Taiwan, which is a social time bomb due to its ageing population. The proportion of the population aged 65 years and above in Taiwan has doubled from 7 % to 1993 to 14 % in 2018 [1], making it an ‘aged society’ according to the World Health Organization. Dementia is a major public health issue in an ageing population. In Taiwan, there are 3.6 million people aged 65 and above, among whom 280,783 have dementia. This implies that for every 12 elderly Taiwanese, there is one person with dementia [2]. Such a high incidence of this disease has a great impact on family life and society. In the modern society, two family members often work full time, and with the prevalence of nuclear families, the demand for long-term care is rising.

Long-term care (LTC) policies are being implemented worldwide in response to the ageing trend [3]. According to the World Bank, LTC financing can be classified into four systems: social insurance models (applied in Netherlands, Germany, Japan, and the Republic of Korea), the universal model (applied in Denmark, Finland and Sweden), means-tested systems (applied in the United Kingdom and the United States), and hybrid systems and approaches (applied in France) [4]. LTC financing in Taiwan is similar to the universal model (tax-based universal public LTC coverage), although a national LTC insurance system can also be built once the development of the service system is complete [5].

Providing care to the rising number of people with dementia remains a public health challenge for health systems worldwide. To tackle these issues, the Taiwanese government has devised a series of LTC-related policies, including the LTC 10-year plan version 1 (LTC 1.0) launched in 2007, and the reformed LTC plan version 2.0, launched in 2016 [6]. LTC 2.0 expanded the community-based and institutional residential dementia care resources already stated by the LTC 1.0 to include dementia patients over 50 years of age as a service target. Moreover, LTC 2.0 also proposed the creation of integrated dementia day care centres that connect services and potential users through case management. The use of different types of care offered to people with dementia may have changed due to the roll-out of the LTC 2.0 reforms, with a lack of empirical evidence regarding the actual use of these services.

Home- and community-based services (HCBS) and support are being increasingly recognised as key components of high-quality dementia care [7]. HCBS is designed to supplement the inadequacy of informal care and assist with the care of home-bound or low-income elderly people [8]. Although the usage of HCBS by older adults who require LTC has been studied [9], service use might be influenced by differences in healthcare delivery systems and cultures [9, 10]. Frequent challenges encountered by people with dementia and their caregivers in using HCBS include limited awareness of dementia and lack of information about available services [11, 12]. Previous studies on dementia care have mainly focused on the use of healthcare service and the cost of these services, as the primary resource of analysing utilisation outcomes [13, 14]. To our knowledge, no study has explored the patterns of HCBS usage by elderly patients with dementia in Taiwan. Empirical knowledge of HCBS utilisation is essential to avoid insufficient and inefficient policies. Hence, examining and understanding their current use is essential.

The purpose of this study was to explore the use of HCBS and the factors influencing its usage from the perspective of patients with dementia in Changhua City, Taiwan. This study attempted to resolve the current information gap by (1) examining the patterns of usage of HCBS provided for LTC, (2) determining the frequency and combinations of HCBS utilisation, and (3) identifying the important factors affecting the use of these services. Understanding HCBS utilisation, including the frequency and combination of services used is necessary to inform about the care needs of the dementia population, such as needs specific to dementia subtypes, as well as for guiding LTC resource allocation.

## Methods

### Data source and study design

This retrospective cross-sectional study used data from a self-developed, integrated dementia care programme, a database from the Changhua Christian Hospital (CCH), maintained by the Changhua Christian Hospital, and Ministry of Health and Welfare in Taiwan. Diagnostic and Statistical Manual of Mental Disorders (DSM-V) criteria were used to diagnose patients with dementia [15]. All assessments were completed by an integrated team, which included physicians, psychologists, social workers, dieticians, occupational therapists, pharmacists, and nursing case managers. Patients diagnosed with dementia were recruited as the study subjects for analysis. After the diagnosis of dementia, the team conducted face-to-face interviews with community residents and their care partners, and after evaluation of each case, a personalised care plan was devised. Follow-up telephonic interviews were conducted mainly by the nursing case manager. If the participants were unable to answer questions independently, a family member provided them with assistance or answered on their behalf. The assessment and follow-up interview processes have been previously described [16].

### Ethical considerations

This study being a retrospective chart review, the team-based assessment information was used for analysis, and informed consent was not obtained since this study used de-identified data with secondary data analysis. The study protocol was approved by the Human Research Protection Program of Changhua Christian Hospital and adhered to the principles of the Declaration of Helsinki (approval no.: 160615; 200128).

### Settings and participants

In this study, we analysed a LTC dataset containing records of patients with dementia living in a central city, in one of Taiwan’s six metropolises. The records contain details of the assessment initially needed and reassessments by telephone interviews every 6 months. During this follow-up process, health indicators and service usage of the care recipient were recorded. The original study was of an open cohort design, and as of September 2018, the research dataset contained records of 822 dementia patients who underwent preliminary evaluations during 2015–2018. The data was cleaned to exclude patients who did not attend reassessments of HCBS use by telephone (*n* = 38) and those living in care facilities (*n* = 47), leaving a total of 737 patients who met the study criteria. Out of the 737 selected patients, 244 patients had used social services during the follow-up period. It was determined from the telephone assessment process that these dementia patients had used HCBS after diagnosis.

### Measurements

#### Independent Variables

In the LTC dataset, case assessment records included baseline sociodemographic variables, and physiological and psychological functions. Participants’ age, sex, marital status, and caregiver were recorded as demographic data. Physiological functions were evaluated according to hypnotic use (yes or no), cardiovascular disease (yes or no), cerebrovascular accident (yes or no), and dependency status. Dependency status was assessed using the International Classification of Functioning (ICF)-based measurement of functioning (0 - no problem, 1 - mild problem, 2 - moderate problem, 3 - severe problem, 4 - complete problem) [17]. Psychological functions were determined on the basis of an affirmative response (yes) to emotional, behavioural, or psychological questions regarding whether the patient had any one of the following conditions during assessment with patients and their caregivers: emotional problems (i.e. crying, fear, dysthymic, depressive, anxiety, anger, emotional liability, apathy, worry, fidgety), or behavioural or psychological problems (agitation, akathisia, wandering, curse, shadowing, aggression, akinetic, stereotype, abnormal circadian rhythm).

#### Outcome Variables

HCBS data were collected from the cohort entry date to the end of 2018, and information on service usage was reported during the need assessment [16]. HCBS resources included the following: home services (such as laundry, housework, and kitchen-based tasks); respite care (in-home, out-of-home, and overnight care); home nursing care; community care centres; home- and community-based rehabilitation; assistive devices; adult day care; adult foster care; home meal delivery; transportation services; mobile shower/bath services; support care for caregivers (social activities such as lectures, outreach, consultation, relief travel, and fraternity); barrier-free environments; and ‘School of Wisdom’ (group cognitive training courses). The use of each service was categorised, with service use set to 1 (used) or 0 (not used). The amount of service usage for an individual was calculated based on the recorded resources (the same service was calculated only once). The scope of service usage was scored from to 1–14; the higher the score, the more services were used.

### Data analyses

**Fig. 1:**
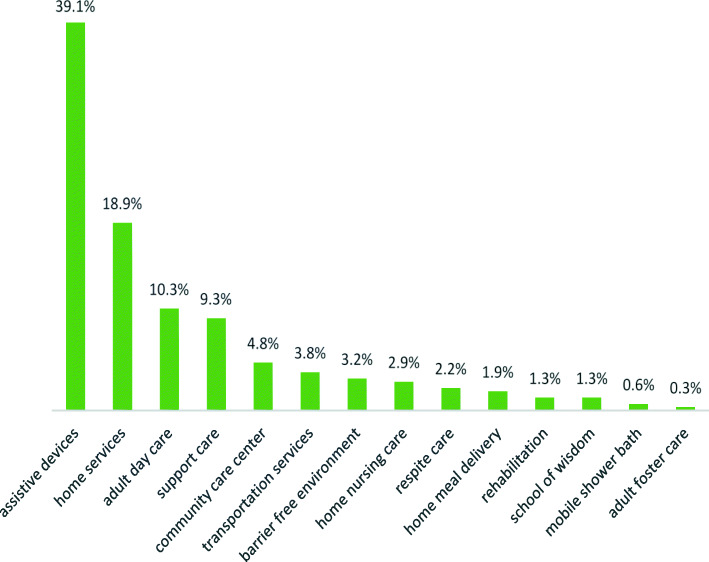
Patterns of service utilisation. Proportion of each service use in the long-term care system throughout the study period. (*n* = 244)

**Fig. 2:**
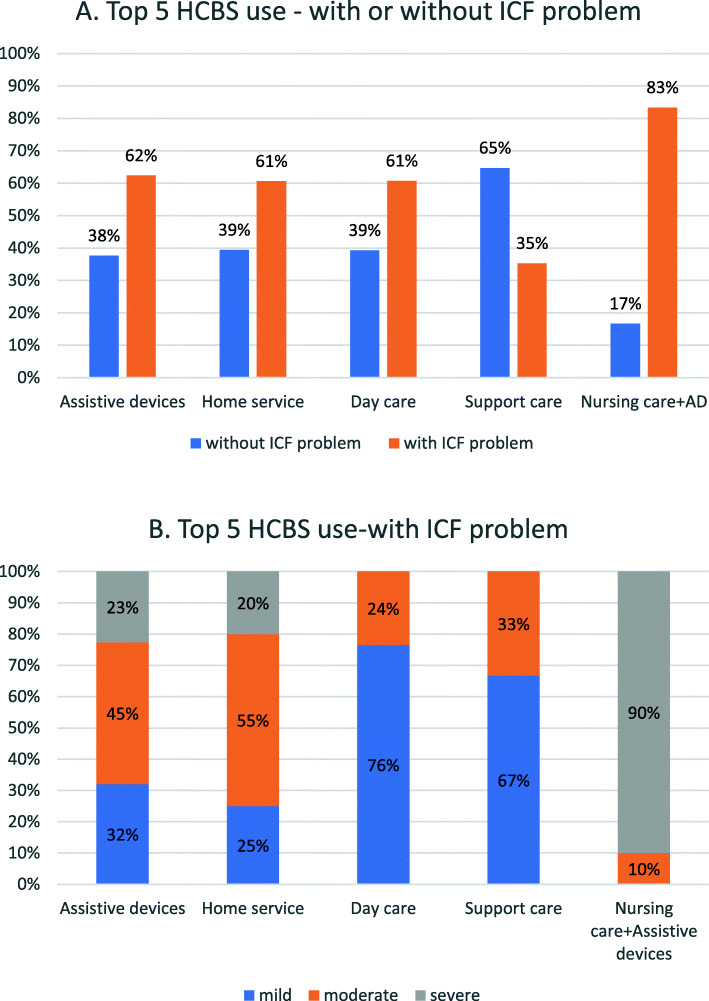
**a**. Percentage of participants with ICF-related or non-ICF related problems by the top 5 home- and community-based services used. **b**. Percentage of service utilisation by dementia-related top 5 HCBS used by participants with mild, moderate and severe ICF problems. (ICF: International Classification of Functioning, Disability and Health)

Descriptive analyses (mean, frequencies, and percentages) were conducted on demographic and clinical data to characterise the sample. A t-test was conducted to compare mean age. The relationships between categorical variables were assessed using the chi-square test. Patients who used ≥ 1 resource were compared to non-users (Table 1). The service use patterns are shown in Fig. 1. The goal of this study was to analyse the different individual effects of independent variables on one dependent variable, the ultimate dependent variable of interest being the count of usage of social services. As it is a count variable and not normally distributed, the Poisson regression model [18, 19] was employed to estimate the number of HCBS used (discrete count outcomes) by patients with dementia. Since the variance (0.478) and the mean (0.420) are equal, implying that the dispersion statistic equals one, a robust Poisson model was used to estimate the regression parameters. Since the service observation period for each participant was different, we added the number of telephone visits (reassessment) in the study period as an offset variable to adjust for total service usage[18–20] (Table 2). We further incorporated service use as service combinations to assess whether service usage was different at different levels of dependency (Fig. 2). IBM SPSS Statistics for Windows, version 22.0 (IBM Corporation, Armonk, NY, USA) was used to analyse the results. The level of significance was set at *p* < 0.05.

**Table 1:** Characteristics of the study group by service use (*N* = 737)

		Health care service					
		Non-users		Users		*p* value	
		*n* = 493	%	*n* = 244	%		
Age (years) Age	mean ± sd <=70	77.88 90	± 8.52 18 %	81.41 18	± 7.81 7 %	*** ***	
	71–80	166	34 %	73	30 %		
	≥ 81	237	48 %	153	63 %		
Sex	female	320	65 %	151	62 %	0.421	
	male	173	35 %	93	38 %		
Marital status	married	288	58 %	127	52 %	0.101	
	divorced/widowed/separated	205	42 %	117	48 %		
Caregiver	myself	45	9 %	17	7 %	0.114	
	spouse/partner	167	34 %	70	29 %		
	children	151	31 %	72	30 %		
	relatives/friends	58	12 %	32	13 %		
	foreign worker/nursing care	72	15 %	53	22 %		
Hypnotics	no	384	78 %	161	66 %	**	
	yes	109	22 %	83	34 %		
CVD	no	430	87 %	211	86 %	0.880	
	yes	47	10 %	24	10 %		
CVA	no	422	86 %	191	78 %	**	
	yes	55	11 %	44	18 %		
Dependency status	without ICF problems	338	69 %	94	39 %	***	
	mild	91	18 %	52	21 %		
	moderate	38	8 %	57	23 %		
	severe	26	5 %	41	17 %		
Emotional problems	no	242	49 %	119	49 %	0.935	
	yes	251	51 %	125	51 %		
Behaviour or psychological problem	no	304	62 %	121	50 %	**	
	yes	189	38 %	123	50 %		

Notes: * *p* < 0.05; ** *p* < 0.01; *** *p* < 0.001; *CVD* cardiovascular disease, *CVA* cerebrovascular accident, *ICF* International Classification of Functioning, Disability and Health

**Table 2:** Factors influencing HCBS utilisation as determined by generalised linear regression analyses (*N* = 737)

		Exp(B)		95 % CI				*p* value
				Lower		Upper		
Age (≤ 70)	≥ 81	1.83	(	1.09	━	3.05	)	*
	71–80	1.69	(	1.03	━	2.78	)	*
Sex (female)	male	1.02	(	0.73	━	1.43	)	0.912
Marital status (married)	unmarried	0.75	(	0.53	━	1.08	)	0.121
Main caregiver (myself)	foreign worker	0.67	(	0.36	━	1.25	)	0.204
	relatives/friends	1.02	(	0.53	━	1.97	)	0.946
	children	0.87	(	0.48	━	1.56	)	0.638
	spouse/partner	0.56	(	0.31	━	1.00	)	0.051
Hypnotics (no)	yes	1.10	(	0.79	━	1.53	)	0.587
CVD (no)	yes	1.27	(	0.83	━	1.93	)	0.268
CVA (no)	yes	1.46	(	0.97	━	2.20	)	0.068
Dependency status (without ICF problems)	severe	3.18	(	2.00	━	5.05	)	***
	moderate	2.62	(	1.67	━	4.10	)	***
	mild	1.69	(	1.15	━	2.49	)	**
Emotional problems (no)	yes	0.84	(	0.59	━	1.18	)	0.314
Behaviour or psychological	yes	1.22	(	0.88	━	1.70	)	0.233
problem (no)								

Notes: * *p* < 0.05; **< 0.01; *** *p* < 0.001; *CI* confidence interval; *CVD* cardiovascular disease, *CVA* cerebrovascular accident, *ICF* International Classification of Functioning, Disability and Health

## Results

### Basic information on Social Services Utilisation

Of the 244 HCBS users, 55.3 % of the patients with dementia used services within 365 days after the initial diagnosis, and the remaining (44.7 %) started to use services after 1 year (data not shown). The results shown in Table 1 indicate that most patients with dementia were older than 81 years. The average age of those who did and did not utilise HCBS was 81 years and 78 years, respectively. In addition, 62–65 % of the patients were female, 52–58 % had a spouse, and 59–65 % lived with their family. Compared with non-users, patients who used HCBS had significantly impaired physical and psychological functions. Finally, the use of services was significantly corelated to the ICF-based measurement of functioning, with 61 % of users having mild to severe problems.

### Pattern of Service Utilisation

Figure 1 presents a description of each service used. For 244 users, variance and mean (SD) of the frequency distribution were 0.350 and 1.28 (± 0.592), respectively. The most frequently used services were assistive devices (39.1 %), followed by home services (18.9 %), adult day care (10.3 %), supportive care for caregivers (9.3 %), community care centres (4.8 %), transportation services (3.8 %), barrier-free environments (3.2 %), home nursing care (2.9 %), respite care (2.2 %), home meal delivery (1.9 %), home- and community-based rehabilitation (1.3 %), school of wisdom (1.3 %), mobile shower/bath services (0.6 %), and adult foster care (0.3 %). In total, this study found that 78 % of patients utilised one service, 17 % utilised two services at the same time, and 4 % utilised three services, whereas only 1 % utilised four services. Overall, the majority of patients with dementia tended to use one service, with only 20 % using multiple services.

### Factors Influencing Service Utilisation

Table 2 shows the factors that affected the use of services by patients with dementia (n = 737). Age was positively correlated with the number of services used, as patients who were older used more services. The adjusted risk ratio (RR) of service ranged from 1.69 (95 % CI = 1.03–2.78) in patients aged 71–80 to 1.83 (95 % CI = 1.09–3.05) in patients aged ≧ 81. The number of services used by patients with dementia increased with the severity of dependence. Compared with patients without problems according to the ICF-based assessment, the adjusted RR of service use ranged from 1.69, in patients with mild dependency, to 2.62, in those with moderate dependency, and to 3.18, in patients with severe dependency. These results imply that the most important factor influencing service usage is dependency status.

### Services used by Dependency Status

Figure 2a-b displays the percentage of dementia-related dependency status by the top 5 HCBS utilisation categories (assistive devices, home service, day care, support care, nursing care and assistive devices). In most types of HCBS, the majority of services used were attributed to ICF problems. This was especially relevant in the case of assistive devices, which were used mainly by patients with moderate disability (45 %), followed by mild disability (32 %), and severe disability (23 %). In addition, people with mild dependency used more day care services (76 %) and support care (67 %) than those with moderate dependency, the majority of whom used more home services (55 %), and those with severe dependency, that mainly used assistive devices in combination with nursing care (90 %). Accordingly, patients with dementia with mild or no problems according to ICF-based assessment were more likely to use community support services, whereas those with moderate to severe dependency were more likely to use in-home care services.

## Discussion

The purpose of this study was to examine patterns of HCBS usage among community-dwelling patients with established dementia diagnoses. The rate of community services used by people with dementia was not as high as expected (33 %), and 78 % of users only used one service, mainly assistive devices. Dementia with disability was the main determinant of most utilisation. The severity of dependency affects the use of services, implying that more service resources may be used by patients with dementia with concomitant severe dependency. This research has made an important contribution to our understanding of the use of care support service systems for patients with dementia residing in the community.

Ageing populations with a growing incidence of disabilities and dementia are important factors driving the increased demand for LTC. LTC 1.0 compiled policies mainly designed for disabled people, whereas services for people with mild to moderate dementia were lacking. Therefore, LTC 2.0 expanded services for people with dementia to eliminate this service gap. Based on the 8 % prevalence rate of dementia in Taiwan, more than 260,000 people suffer from dementia; however, only 30 % of them are properly diagnosed and treated accordingly [21]. This study shows that the utilisation rate of LTC services for the elderly with dementia was 33 %. Since the goal of the LTC plan is to build a powerful and accessible service system, the coverage is clearly insufficient. The dementia services provided by LTC are limited to patients who have been diagnosed with dementia [22], resulting in unavailability of needed care to people who have not yet been diagnosed with dementia but have cognitive impairment. This raises the concern about whether there is adequate care for people with early dementia and how to implement the connection services such as subacute care for those with cognitive decline at an early stage.

Supportive services such as assistive technology (AT) devices have been shown to be associated with the time that people with dementia remain in their own homes [23, 24]. The establishment of 10 AT centres in the Changhua area has not only increased the convenience of accessing AT services but has also enhanced the public’s willingness to use AT devices. A recent review found that dementia caregivers find certain types of AT useful, such as GPS trackers, motion sensors, or medication reminders [25]. This trend may reflect changes in basic health conditions (for example, a decrease in the level of disability) and a tendency to shift to the use of equipment rather than human care (i.e., increasing acceptance of technology). It is well known that nearly 50 % of users accept AT devices, and those with moderate to severe dependency had the highest rate of AT use. However, it is not clear whether providing AT alone can maintain the independence of patients or what the actual impact on caregiver burden and satisfaction is. Therefore, in the future it is necessary to address if patients who are highly dependent on AT equipment require other inputs or options.

It is not surprising that dependency status is related to increased LTC utilisation [26]. Dementia is related to age, and even after extensive control for age, disability remains an important driver of LTC use. Interestingly, the use of service resources varies according to the level of dependency. People with mild dementia tend to prefer to use community support services (day care centres), whereas those with moderate to severe dependency tend to prefer to use in-home care services (home care and nursing care). This raises the concern about whether there is adequate care for people with dementia at different levels of disability. The degree of dependence may affect a person’s living arrangements, which in turn may affect choices related to the use of LTC services [27]. A recent study has shown that about 75 % of dementia patients in Western countries are cared for by their family members [28]. Older people with dementia need much more support and protection from their family or society, so older individuals with higher dependency levels are more likely to live with a spouse or adult children. Hence, informing family caregivers’ about the early symptoms of the disease and the existing service resources is essential to facilitate early diagnosis of impaired cognitive function and provide the required services in time.

In contrast to national data on service use in Taiwan [29], home care was not the most commonly used service in this study, and the use of home care was much greater among those with moderate disability. It could be expected that when functional disability is more severe, such as difficulty in walking and eating, the frequency of in-home help usage will be substantially greater. The needs of people with dementia and their caregivers are diverse, and the services that support them do not always meet their needs effectively [30]. Very few studies have been performed on the feelings of dementia caregivers about receiving home services and whether or not their needs are met, and the effect of home services in reducing the burden of caregivers is not yet clear [31, 32]. Accordingly, the expression of care needs and whether the care needs are being met are important indicators that affect LTC service use and are worthy of further discussion.

National LTC policies in different countries are influenced by the specific historic background, politics, resources, culture, and the role of the government in social welfare [3, 33, 34]. The different policies about care provision across countries vary in the way informal care is treated when care needs assessment for publicly funded LTC. For example, in England and Australia, the amount of informal care provided by relatives is taken into account and financed by the government, whereas this is not the case in France or Japan [35]. LTC 2.0 considers the unaffordability of private care (for example, for low-income households) and the unavailability of informal care (for example, for people without family in close proximity), and establishes protocols to meet people’s needs. The current policies are designed to encourage that the elderly stay at their own home for as much time as possible, and therefore, most resources have been allocated to in-home care and community care. Hence, the purpose of HCBS is to supplement the insufficiency of informal care and to support the elderly with community-living or low-income and able to ageing in place [8]. Although the Taiwan policies are distinctly ‘Taiwanese’, all countries worldwide are facing similar challenges now and will need to develop more comprehensive strategies to address this multidimensional challenge.

This study examined a cohort of community-dwelling older adults with dementia using comprehensive telephone interview data from a medical centre in central Taiwan. However, this study had some limitations. As we did not capture the same service during the 2 years of follow-up, the impact of dementia on service utilisation has been underestimated. For example, people who used service A at baseline could use services A and B in the second year, but we only counted the use of two services. Although we could provide a snapshot of the use of dementia-related community services in our cohort, we lacked information on their degree of dementia. As the cognition and physical function of patients degenerate simultaneously during the tracking process, ICF-based assessment of problems was used instead of the degree of dementia as the main distinguishing criterion. In addition, in this research, only the opinions of those using HCBS were collected, whereas the opinions of service providers or those who did not use services were not obtained. Therefore, future research should focus on the views of those who do not use services and compare them with those who do use services. Finally, the dataset was obtained from a medical centre in central Taiwan, and the sample size focused only on those with dementia, diagnosed using a cross-sectional study design; therefore, generalisation of the results should be performed with caution under the LTC policy nationwide.

## Conclusions

This study uses Taiwan as an example to explore the experience of LTC service use. Recognising the diversity of elderly people with dementia, at the level of functional impairment and in their ability to secure help without LTC support, is essential for developing policies that meet the wide range of needs of elderly people. In addition to providing in-home care or community support services based on the degree of dementia and disability, further research should be conducted on the benefits of AT devices. Increasing awareness of service availability is essential to enable caregivers to better match service use with patient needs. To ensure that recipients’ needs are met after enrolling in the publicly funded LTC system, a more accurate protocol to assess the patient’s needs must be developed and implemented policies should encourage all types of service resources to settle in areas currently not covered. This will ensure recipients continue to live in their home communities and receive care, which will achieve the goal of the ageing in place policy.

## Data Availability

The data that support the findings of this study are available from the dementia centre of Changhua Christian Hospital, but restrictions apply to the availability of these data, which were used under licence for the current study, and so are not publicly available. However, data are available from the authors upon reasonable request and with permission from the dementia centre of Changhua Christian Hospital.

## Abbreviations

HCBS: Home- and community-based service
LTC: Long-term care
CCH: Changhua Christian Hospital
DSM-V: Diagnostic and Statistical Manual of Mental Disorders
ICF: International Classification of Functioning
AT: Assistive technology
GPS: Global Positioning System
